# POLICY SERIES: AGING AND PUBLIC POLICY: NAVIGATING THE INTERSECTIONS

**DOI:** 10.1093/geroni/igae098.0306

**Published:** 2024-12-31

**Authors:** Michael Lepore, Tara McMullen, Karon Phillips

**Affiliations:** Chair; Co-Chair; Discussant

## Abstract

Contemporary politics and public policy are rife with matters of aging and inequality. While an impressive history of public health achievements over infectious diseases has enhanced the possibility for populations to enjoy longer and healthier lives, these benefits have not been enjoyed equally. Rather than overcome disparities in longevity and health, existing public policies designed for the younger and less diverse populations of earlier generations have potential to further entrench age-related inequalities in current populations into the future. The Gerontological Society of America’s policy journal, Public Policy & Aging Report, has a rich history of directly tackling the thorny political issues at the heart of these inequalities, including national voting patterns of older White Americans supporting conservative political candidates and values that clash with their support of policies and programs from which they directly benefit. This symposium draws on such historic trends and contemporary research to shed light on the intersections of aging and public policy that we are navigating today, including federal and state legislative and regulatory initiatives to improve the economic security of low-income older adults, local programs in numerous states that use property tax levies for social care services to assist in maintaining community independence, and issues of voting access in the context of aging, disability, and cognitive impairment. Contributors to this session will build on recent publications in Public Policy & Aging Report and will engage in discussion addressing the 2024 US election and looking ahead to the future of aging and public policy.

